# Therapeutic Notes

**Published:** 1907-08-17

**Authors:** 


### THERAPEUTIC NOTES.

### The Price of Camphor.

It was pointed out in these columns some weeks ago that in view of the declining value of crude camphor it was difficult to understand how the high price of the refined article used in medicine could be maintained, and it was indicated that a reduction might be expected. During the last few weeks Japanese refined camphor has, in fact, been gradually diminishing in value, and English refined has now followed suit. The reduction made by refiners is 11d. per pound, which may be considered substantial, although the price now quoted is still considerably above that which rules when free supplies of the raw material are obtainable from the producing district. Any circumstance which would tend to lessen the cost of a useful preparation like liniment of camphor is to be welcomed, and it is to be hoped that the present reduction of 11d. will be followed by further price alterations in a similar direction.

